# Supplementary material for: HIVprotI: an integrated web based platform for prediction and design of HIV proteins inhibitors
Source: J Cheminform. 2018 Mar 9;10:12. doi: 10.1186/s13321-018-0266-y (PMC5845081; doi:10.1186/s13321-018-0266-y)
Supplement: Supplementary file 2 — Additional file 2. Source code of HIVProtI web server. [file 13321_2018_266_MOESM2_ESM.zip › HIVprotI_Source-code/hivprotI/weka-3-6-6/doc/weka/gui/beans/ConnectionNotificationConsumer.html]

ConnectionNotificationConsumer


---


|  |  |  |
| --- | --- | --- |
| **Overview** \| **Package** \| **Class** \| **Tree** \| **Deprecated** \| **Index** \| **Help** \| **Weka's home** | |  |
| **PREV CLASS**   **NEXT CLASS** | **FRAMES**    **NO FRAMES**     **All Classes** |  |
| SUMMARY: NESTED \| FIELD \| CONSTR \| METHOD | DETAIL: FIELD \| CONSTR \| METHOD |  |


---


## weka.gui.beans Interface ConnectionNotificationConsumer

---

``` public interface ConnectionNotificationConsumer ```

Interface for Beans that can receive (dis-)connection events generated when
(dis-)connecting data processing nodes in the Weka KnowledgeFlow.
This is useful, for example, for "intelligent" filters that are able to share
configuration information with preceding nodes in the processing chain.

**Version:**
:   $Revision: 1.2 $

**Author:**
:   Carsten Pohle (cp AT cpohle de)

---

| **Method Summary** | |
| --- | --- |
| `void` | `connectionNotification(java.lang.String eventName, java.lang.Object source)`             Notify this object that it has been registered as a listener with a source with respect to the supplied event name. |
| `void` | `disconnectionNotification(java.lang.String eventName, java.lang.Object source)`             Notify this object that it has been deregistered as a listener with a source with respect to the supplied event name This method should be implemented synchronized. |

**Method Detail**

### connectionNotification

```
void connectionNotification(java.lang.String eventName,
                            java.lang.Object source)
```

:   Notify this object that it has been registered as a listener with a source
    with respect to the supplied event name.
    This method should be implemented synchronized.

    :   **Parameters:**: `eventName` -: `source` - the source with which this object has been registered as a listener

---


### disconnectionNotification

```
void disconnectionNotification(java.lang.String eventName,
                               java.lang.Object source)
```

:   Notify this object that it has been deregistered as a listener with a
    source with respect to the supplied event name
    This method should be implemented synchronized.

    :   **Parameters:**: `eventName` - the event: `source` - the source with which this object has been registered as a listener


---


|  |  |  |
| --- | --- | --- |
| **Overview** \| **Package** \| **Class** \| **Tree** \| **Deprecated** \| **Index** \| **Help** \| **Weka's home** | |  |
| **PREV CLASS**   **NEXT CLASS** | **FRAMES**    **NO FRAMES**     **All Classes** |  |
| SUMMARY: NESTED \| FIELD \| CONSTR \| METHOD | DETAIL: FIELD \| CONSTR \| METHOD |  |


---
